# Prognostic factors and survival prediction of resected non-small cell lung cancer with ipsilateral pulmonary metastases: a study based on the Surveillance, Epidemiology, and End Results (SEER) database

**DOI:** 10.1186/s12890-023-02722-y

**Published:** 2023-10-30

**Authors:** Jiajun Zhang, Jin Zhang

**Affiliations:** 1 Ningxia Medical University, Yinchuan, 750004 People’s Republic of China; 2 Department of Respiratory and Critical Care Medicine, General Hospital of Ningxia Medical University, 804 Shengli South Street, Xingqing District, Yinchuan, 750004 China

**Keywords:** Non-small cell lung cancer, Ipsilateral pulmonary metastasis, Prognostic factors, Survival outcomes, Nomogram

## Abstract

**Background:**

Prognostic factors and survival outcomes of non-small cell lung cancer (NSCLC) with Ipsilateral pulmonary metastasis (IPM) are not well-defined. Thus, this study intended to identify the prognostic factors for these patients and construct a predictive nomogram model.

**Methods:**

One thousand, seven hundred thirty-two patients with IPM identified between 2000 to 2019 were from the Surveillance, Epidemiology, and End Results (SEER) database. Independent prognostic factors were identified using multivariate Cox regression analyses. Nomograms were constructed to predict the overall survival (OS), C-index, the area under the curve (AUC), and the calibration curve to determine the predictive accuracy and discrimination; the decision curve analysis was used to confirm the clinical utility.

**Results:**

Patients were randomly divided into training (*n* = 1213) and validation (*n* = 519) cohorts. In the training cohort, the multivariable analysis demonstrated that age, sex, primary tumor size, N status, number of regional lymph nodes removed, tumor grade, and chemotherapy were independent prognostic factors for IPM. We constructed a 1-year, 3-year, and 5-year OS prediction nomogram model using independent prognostic factors. The C-index of this model for OS prediction was 0.714 (95% confidence interval [CI], 0.692 to 0.773) in the training cohort and 0.695 (95% CI, 0.660 to 0.730) in the validation cohort. Based on the AUC of the receiver operating characteristic analysis, calibration plots, and decision curve analysis, we concluded that the prognosis model of IPM exhibited excellent performance. Patients with total nomogram points greater than 96 were considered high-risk.

**Conclusion:**

We constructed and internally validated a nomogram to predict 1-year, 3-year, and 5-year OS for NSCLC patients with IPM according to independent prognostic factors. This nomogram demonstrated good calibration, discrimination, clinical utility, and practical decision-making effects for the prognosis of NSCLC patients with IPM.

## Introduction

Ipsilateral pulmonary metastasis (IPM), also known as separate or additional tumor nodule(s), is a form of intrapulmonary metastasis in non-small cell lung cancer (NSCLC), including tumors with additional nodule(s) in the same lobe (PM1) and tumors with additional nodule(s) in the ipsilateral different lobe (PM2) [1]. The incidence of IPM in NSCLC ranges from 3–10%, with PM1 occurring in 2–7% of cases and PM2 occurring in 1–3% [2–6]. Since the latest classification change for PM in the 7th edition American Joint Committee on Cancer (AJCC) tumor-node-metastasis (TNM) staging system, PM1 and PM2 were reclassified as the descriptor of T3 and T4 from T4 and M1, respectively [1].

Although the current AJCC staging system still relies on the location relationship between IPM and the primary tumor [7], previous studies have demonstrated that prognostic factors of IPM are not solely related to its location [3–6, 8]. In recent years, there has been a rise in both surgical and systemic adjuvant therapy for patients with IPM [9–11]. Research has shown that NSCLC patients with IPM tend to have a better prognosis compared to those with other descriptors of the T3 or T4 stage [12, 13]. Some recent studies have recommended further downstaging of NSCLC patients with IPM [14, 15]. Therefore, studying the survival outcome and prognostic factors of these patients after surgery remains critical.

Nomogram is a convenient and accurate method to predict survival outcomes, which has become very popular in malignant tumor survival prediction in recent years [16]. While many predictive models for extrapulmonary metastasis in NSCLC have been established, and their accuracy and discrimination had been validated by internal and external data [17–19], there is a dearth of research on intrapulmonary metastasis with predictive models, and the sample size of prognostic studies for these patients is relatively small. To fill this gap, we plan to construct a nomogram that can accurately predict the survival of these patients using data from the Surveillance, Epidemiology, and End Results (SEER) database.

## Methods

### Data sources

One thousand, seven hundred thirty-two patients with IPM were identified between 2000 to 2019 from the database "Incidence – SEER Research Plus Data, 17 Registries, Nov 2021 Sub (2000–2019)”. The SEER database was publicly available and established in 1973. The database currently collects and publishes cancer incidence and survival data from population-based cancer registries covering approximately 48.0% of the U.S. population. Data from the SEER database includes demographic and follow-up information.

### Patient selection

The inclusion criteria were: (1) primary lung cancer; (2) tumor descriptor with ipsilateral pulmonary metastasis [based on Separate Tumor Nodules Ipsilateral Lung Recode (2010 +) with the information of separate nodules of the same hist type in the ipsilateral lung, same or different lobe]; (3) no overlapping primary sites; (4) histological confirmation of adenocarcinoma (histologic codes 8244, 8245, 8250–8255, 8260, 8290, 8310, 8323, 8333, 8480, 8481, 8490, 8507, 8550, 8570, 8571, 8574, and 8576), squamous cell carcinoma (histologic codes 8052, 8070–8075, 8083, 8084, 8123), large cell carcinoma (histologic codes 8012–8014), and other non-small-cell carcinoma (8046, 8050, 8003,8004, 8022, 8031–8035, 8082, 8200, 8240, 8249, 8430, 8560, 8562, 8980); (6) history of surgery (sublobectomy, lobectomy or pneumonectomy); and (7) survival time should be at least one month.

**Fig. 1:**
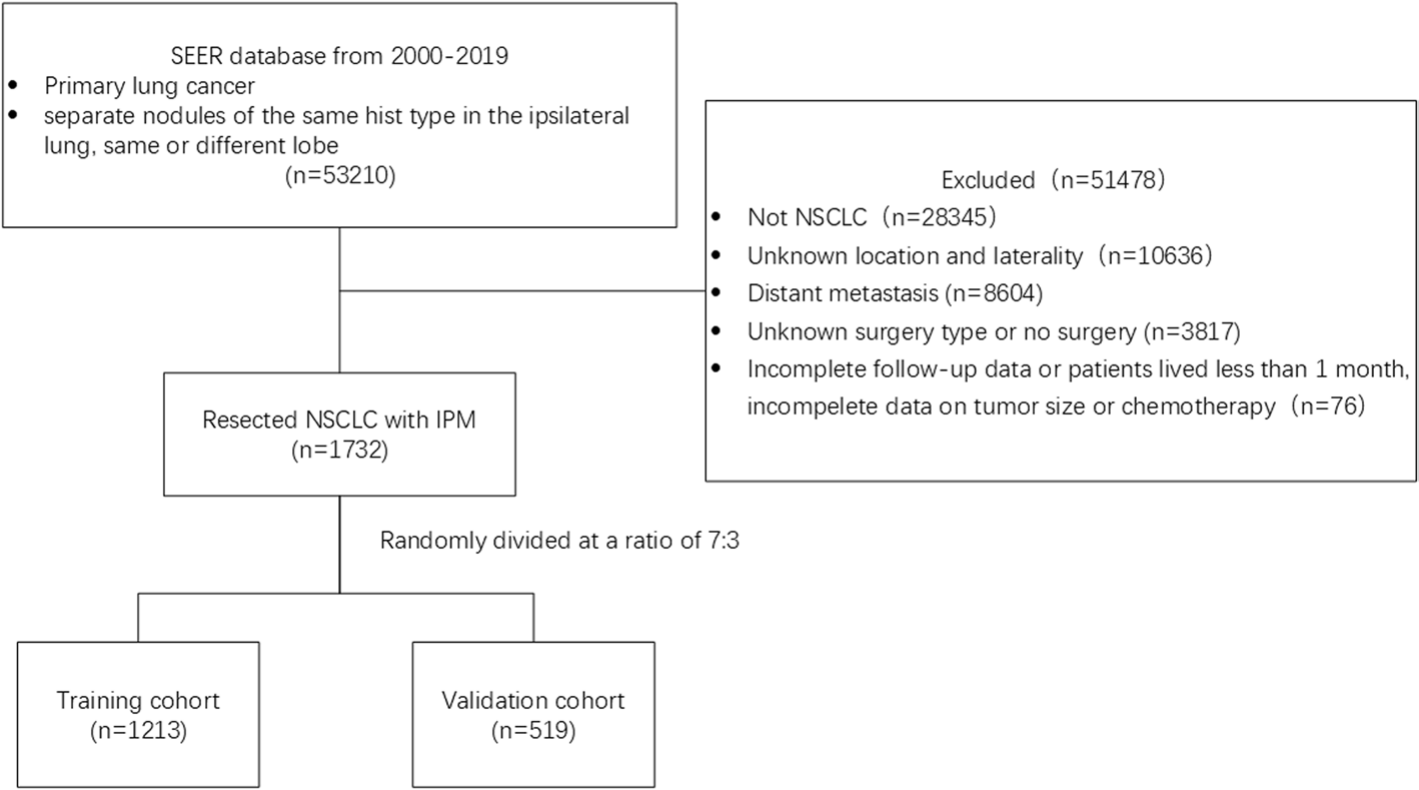
Flow diagram of the data extraction process. SEER, the Surveillance, Epidemiology, and End Results; NSCLC, non-small cell lung cancer; IPM, ipsilateral pulmonary metastasis

The exclusion criteria were: (1) unknown tumor location and laterality; (2) without any treatment; and (4) incomplete demographic data. More details of the data extraction process are presented in Fig. 1.

### Statistical analysis

Random division of patients, all analyses and figures were performed by R version 4.2.2 and R studio 2022.12.0 Build 353 (https://www.r-project.org/). Pearson’s chi-squared test compared categorical variables of clinical features of the patients. Cox regression analysis was used to further investigate the factors affecting the overall survival (OS). The multivariate analyses enrolled the statistically significant variables whose *P* value < 0.1 from univariate analyses. A nomogram was constructed based on the risk factors identified from the multivariate analysis in R studio to predict the 1-year, 3-year, and 5-year OS. The performance of the model was measured using the concordance index (C-index), calibration curves, receiver operating characteristic (ROC) curve, the area under the ROC curve (AUC), and decision curve analysis (DCA). According to the cutoff of the nomogram score, the samples were divided into a high-risk group and a low-risk group. Survival curves were constructed using the Kaplan–Meier method and compared using the log-rank test. During the internal validation of the nomogram, the C-index, calibration curves, ROC curves, and DCA curves were derived from the regression analysis in R. Statistical significance was assumed at a two-sided probability value < 0.05. Patient data were extracted by SEER*Stat software, version 8.4.0.1.

## Results

### Baseline characteristics of patients

From 2000 to 2019, 1732 resected NSCLC patients with IPM were enrolled, 1334 (77%) had PM1 and 398 (23%) had PM2. The median age of the patients was 69 years, 274 patients were male, and the median tumor size was 27 mm. For the tumor histology, 588 patients were diagnosed with squamous cell cancer (SCC), 730 patients were diagnosed with adenocarcinoma (ADC), and 414 patients were diagnosed with other types of NSCLC. Moreover, chemotherapy was administered to 751 patients, and radiotherapy was administered to 290 patients. Regarding the surgery, 348 patients underwent sublobectomy,1287 patients underwent lobectomy, and the rest 97 patients underwent pneumonectomy. All enrolled patients were randomly divided into a training cohort (*n* = 1213, 70%) and a validation cohort (*n* = 519, 30%). No significant differences were found in variables between the two cohorts (*P* > 0.05). A detailed comparison of all enrolled patients' characteristics is presented in Table 1.

**Table 1:** Characteristics of NSCLC with ipsilateral pulmonary metastasis

Variables	All patients (*n* = 1732)		Training cohort (*n* = 1213)	Validation cohort (*n* = 519)	*P* value
Age(%)					0.646
≤ 65	635	(36.7)	440 (36.3)	195 (37.6)	
> 65	1097	(63.3)	773 (63.7)	324 (62.4)	
Sex(%)					0.543
Male	932	(53.8)	659 (54.3)	273 (52.6)	
Female	800	(46.2)	554 (45.7)	246 (47.4)	
Race(%)					0.171
White	1484	(85.7)	1042 (85.9)	442 (85.2)	
Black	130	(7.5)	83 (6.8)	47 (9.1)	
Others	118	(6.8)	88 (7.3)	30 (5.8)	
Laterality(%)					0.213
Left	671	(38.7)	482 (39.7)	189 (36.4)	
Right	1061	(61.3)	731 (60.3)	330 (63.6)	
Location(%)					0.889
Upper	944	(54.5)	660 (54.4)	284 (54.7)	
Middle	124	(7.2)	85 (7.0)	39 (7.5)	
Lower	664	(38.3)	468 (38.6)	196 (37.8)	
Primary tumor size(%)					0.281
≤ 3 cm	990	(57.2)	700 (57.7)	290 (55.9)	
3-5 cm	396	(22.9)	284 (23.4)	112 (21.6)	
5-7 cm	197	(11.4)	127 (10.5)	70 (13.5)	
> 7 cm	149	(8.6)	102 (8.4)	47 (9.1)	
Histology(%)					0.952
Squamous cell cancer	588	(33.9)	411 (33.9)	177 (34.1)	
Adenocarcinoma	730	(42.1)	514 (42.4)	216 (41.6)	
other NSCLC	414	(23.9)	288 (23.7)	126 (24.3)	
Grade(%)					0.964
I	298	(17.2)	212 (17.5)	86 (16.6)	
II	560	(32.3)	387 (31.9)	173 (33.3)	
III	488	(28.2)	343 (28.3)	145 (27.9)	
IV	18	(1.0)	12 (1.0)	6 (1.2)	
Unknown	368	(21.2)	259 (21.4)	109 (21.0)	
lpsilateral pulmonary metastasis(%)					0.826
PM1	1334	(77.0)	932 (76.8)	402 (77.5)	
PM2	398	(23.0)	281 (23.2)	117 (22.5)	
N(%)					0.298
N0	1166	(67.3)	822 (67.8)	344 (66.3)	
N1	260	(15.0)	172 (14.2)	88 (17.0)	
N2	284	(16.4)	200 (16.5)	84 (16.2)	
N3	12	(0.7)	11 (0.9)	1 (0.2)	
Nx	10	(0.6)	8 (0.7)	2 (0.4)	
Number of regional lymph nodes removed(%)					0.406
None/unknown	262	(15.1)	175 (14.4)	87 (16.8)	
1–3	211	(12.2)	153 (12.6)	59 (11.4)	
≥ 4	1258	(72.6)	885 (73.0)	373 (71.9)	
Chemotherapy(%)					0.871
No/unknown	981	(56.6)	685 (56.5)	296 (57.0)	
Yes	751	(43.4)	528 (43.5)	223 (43.0)	
Radiotherapy(%)					0.822
None/unknown	1442	(83.3)	1012 (83.4)	430 (82.9)	
Yes	290	(16.7)	201 (16.6)	89 (17.1)	
Surgery(%)					0.892
Sublobectomy	348	(20.1)	245 (20.2)	103 (19.8)	
Lobectomy	1287	(74.3)	898 (74.0)	389 (75.0)	
Pneumonectomy	97	(5.6)	70 (5.8)	27 (5.2)	

*NSCLC* non-small cell lung cancer, *PM1* Tumors with additional nodule(s) in the same lobe, *PM2* Tumors with additional nodule(s) in the ipsilateral different lobe

### Independent prognostic factors in the training cohort

All the variables were included in the Cox regression. After univariate analysis, age, sex, location in middle lobe, primary tumor size, tumor histology, tumor grade, ipsilateral pulmonary metastasis type, N status, number of reginal lymph nodes removed, chemotherapy, radiotherapy and surgery type were selected into the multivariate analysis. Finally, the analysis demonstrated that age, sex, primary tumor size, tumor grade, N status, number of reginal lymph nodes removed and chemotherapy were the independent prognosis factors for OS. The results of the univariate and multivariate analysis are shown in Table 2.

**Table 2:** Cox proportional hazards model for OS of the resected NSCLC with ipsilateral pulmonary metastasis in the training cohort

Variables	Univariate analysis			Multivariate analysis		
	HR	95% CI	P	HR	95% CI	P
Age						
≤ 65	Reference			Reference		
> 65	1.764	1.463–2.126	< 0.001	1.978	1.615–2.421	< 0.001
Sex						
Female	Reference			Reference		
Male	1.845	1.558–2.184	< 0.001	1.501	1.259–1.803	< 0.001
Race						
White	Reference					
Black	1.227	0.900–1.671	0.195			
Others	0.978	0.705–1.356	0.894			
Laterality						
Left	Reference					
Right	0.934	0.788–1.107	0.432			
Location						
Upper	Reference			Reference		
Middle	0.709	0.484–1.038	0.077	0.907	0.612–1.345	0.629
Lower	1.076	0.904–1.280	0.409	1.132	0.945–1.356	0.178
Primary tumor size						
≤ 3 cm	Reference			Reference		
3-5 cm	1.703	1.396–2.079	< 0.001	1.383	1.114–1.719	0.003
5-7 cm	1.797	1.387–2.328	< 0.001	1.451	1.094–1.925	0.010
> 7 cm	2.676	2.038–3.513	< 0.001	2.230	1.650–3.013	< 0.001
Histology						
Squamous cell cancer	Reference			Reference		
Adenocarcinoma	0.575	0.477–0.693	< 0.001	0.915	0.744–1.126	0.402
other NSCLC	0.539	0.429–0.677	< 0.001	0.945	0.945–1.356	0.657
Grade						
I	Reference			Reference		
II	2.028	1.520–2.706	< 0.001	1.762	1.298–2.391	< 0.001
III	3.068	2.308–4.078	< 0.001	2.273	1.660–3.112	< 0.001
IV	3.587	1.781–7.224	< 0.001	3.537	1.706–7.332	< 0.001
Unknown	1.429	1.017–2.009	0.040	1.238	0.869–1.762	0.237
lpsilateral pulmonary metastasis						
PM1	Reference			Reference		
PM2	1.256	1.040–1.516	0.018	1.067	0.873–1.305	0.526
N						
N0	Reference			Reference		
N1	1.888	1.498–2.380	< 0.001	1.640	1.276–2.106	< 0.001
N2	2.333	1.903–2.861	< 0.001	2.032	1.597–2.584	< 0.001
N3	9.723	5.132–18.420	< 0.001	7.273	3.687–14.349	< 0.001
Nx	1.874	0.698–5.026	0.212	1.668	0.604–4.606	0.324
Number of regional lymph nodes removed						
None/unknown	Reference			Reference		
1–3	0.787	0.577–1.074	0.131	0.725	0.519–1.012	0.059
≥ 4	0.813	0.647–1.022	0.076	0.643	0.485–0.851	< 0.001
Chemotherapy						
No/unknown	Reference			Reference		
Yes	1.336	1.131–1.580	0.001	0.805	0.652–0.994	0.044
Radiotherapy						
None/unknown	Reference			Reference		
Yes	1.802	1.481–2.194	< 0.001	1.183	0.936–1.497	0.160
Surgery						
Sublobectomy	Reference					
Lobectomy	0.925	0.750–1.140	0.464			
Pneumonectomy	1.338	0.938–1.907	0.108			

*NSCLC* non-small cell lung cancer, *OS* overall survival, *HR* hazard ratio, *CI* confidence interval, *PM1* Tumors with additional nodule(s) in the same lobe, *PM2* Tumors with additional nodule(s) in the ipsilateral different lobe

### Construction and validation of nomogram for predicting OS

**Fig. 2:**
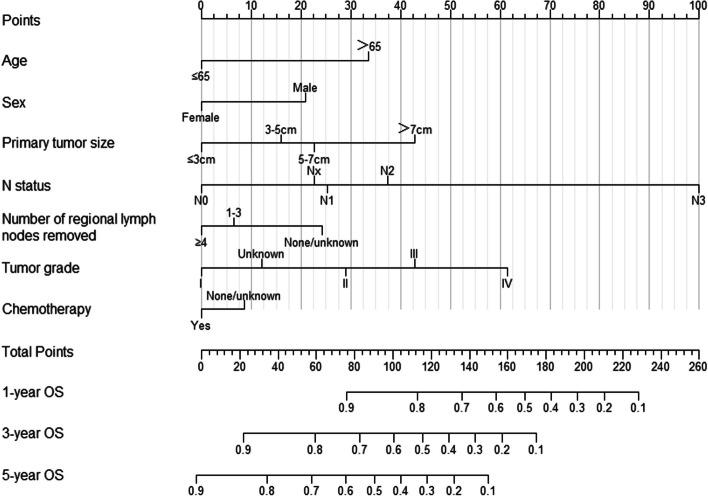
Nomogram for predicting OS of NSCLC patients with IPM. OS, overall survival; NSCLC, non-small cell lung cancer; IPM, ipsilateral pulmonary metastasis

Based on the results of the multivariate Cox regression model, a nomogram was constructed using independent factors including age, sex, primary tumor size, tumor grade, N status, number of regional lymph nodes removed, and chemotherapy. The nomogram can predict the 1-year, 3-year, and 5-year OS rates by summing the points of each variable (Fig. 2).

**Fig. 3:**
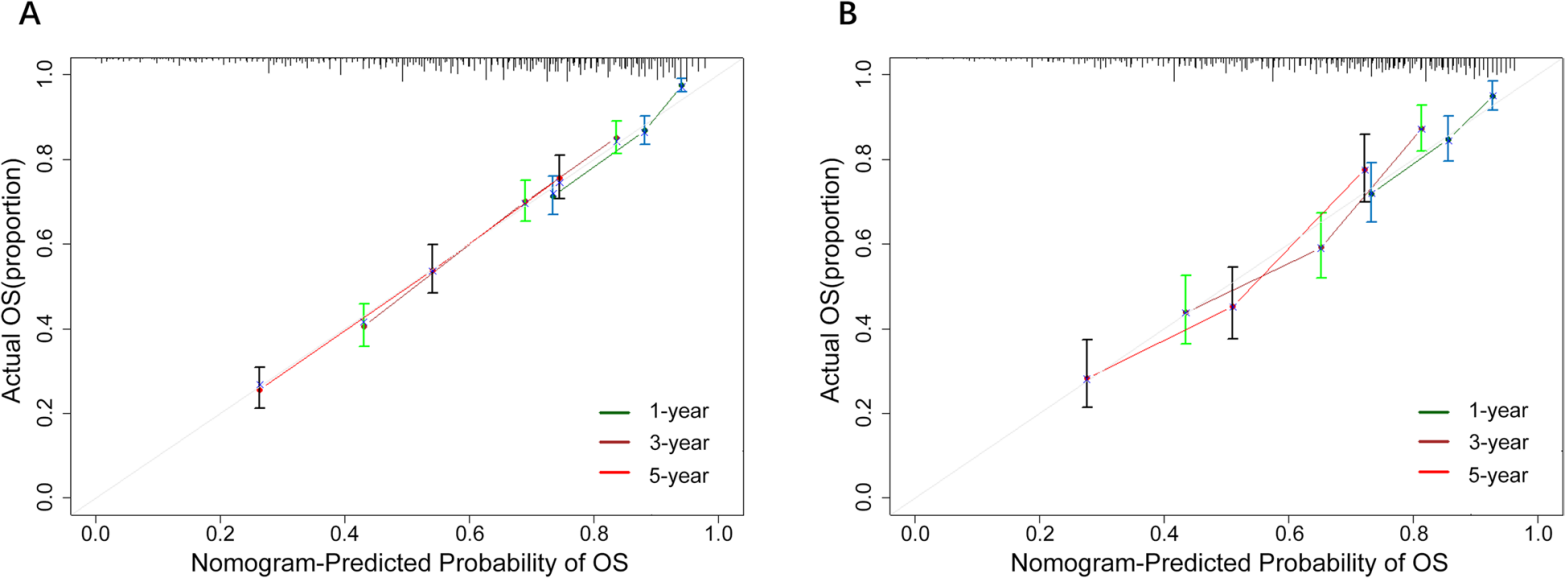
The calibration curve to evaluate the accuracy of the nomogram at 1, 3 and 5 years, respectively. **A** The calibration curve analysis of the nomogram compared for 1, 3, and 5 years in the training cohort. **B** The calibration curve analysis of the nomogram compared for 1, 3, and 5 years in the validation cohort

The C-index for OS prediction was 0.714 (95% CI, 0.692 to 0.773) in the training cohort and 0.695 (95% CI, 0.660 to 0.730) in the validation cohort, indicating good discrimination ability. The calibration plots for the probability of survival at 1, 3, and 5 years showed good agreement between nomogram-predicted OS and actual observation (Fig. 3A, B).

**Fig. 4:**
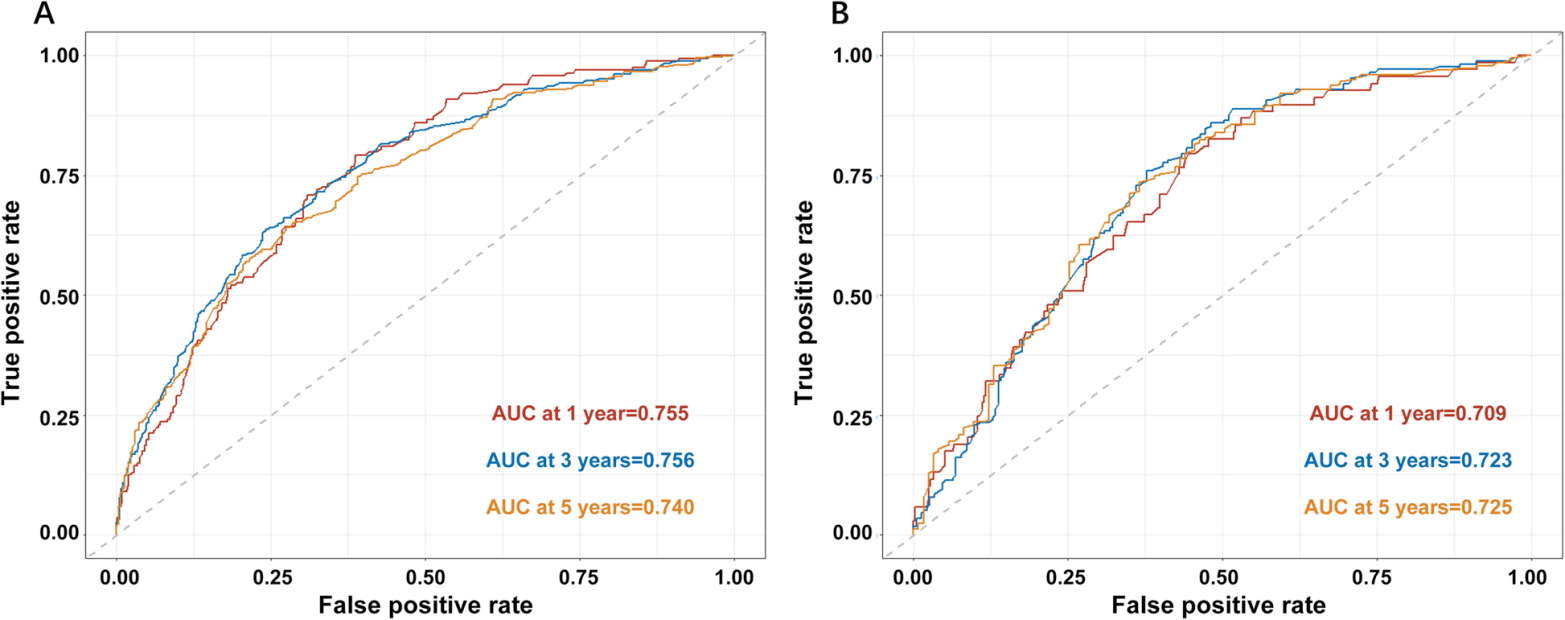
The AUC values for the prediction of 1, 3, 5-year OS of NSCLC patients with IPM. **A** in the training cohort. **B** in the validation cohort. AUC, the area under the curve; OS, overall survival; NSCLC, non-small cell lung cancer; IPM, ipsilateral pulmonary metastasis

The ROC analysis assessed the accuracy of the nomogram, and the AUCs for predicting 1-year, 3-year, and 5-year OS rates in the training cohort were 0.755 (95% CI, 0.724 to 0.786), 0.756 (95% CI, 0.730 to 0.783), and 0.740 (95% CI, 0.710 to 0.770), respectively. In the validation cohort, the AUCs for predicting 1-year, 3-year, and 5-year OS rates were 0.709 (95% CI, 0.657 to 0.761), 0.723 (95% CI, 0.682 to 0.764), and 0.725 (95% CI, 0.676 to 0.774), respectively (Fig. 4A, B), indicating good discriminative ability of the nomogram.

**Fig. 5:**
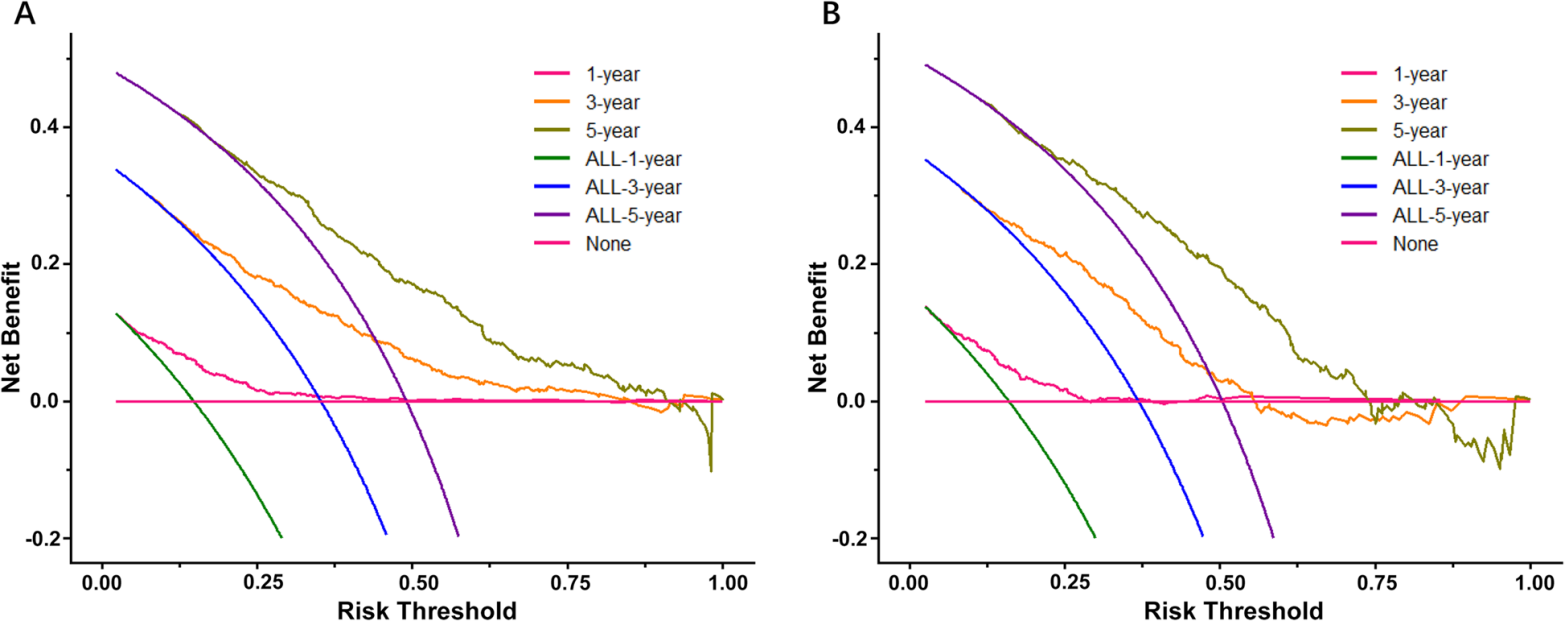
The DCA curve analysis to evaluate the clinical application ability of the nomogram at 1, 3 and 5 years, respectively. **A** The DCA curve analysis of the nomogram compared for 1, 3, and 5 years in the training cohort. **B** DCA curve analysis of the nomogram compared for 1, 3, and 5 years in the validation cohort

The DCA demonstrated that the nomogram had a significant clinical value in both the training and validation cohorts. The area under the DCA curve for predicting 1-year, 3-year, and 5-year OS rates in the training cohort was 0.017, 0.078, and 0.142, respectively. In the validation cohort, the area under the DCA curve was 0.017, 0.084, and 0.148 (Fig. 5A, B).

**Fig. 6:**
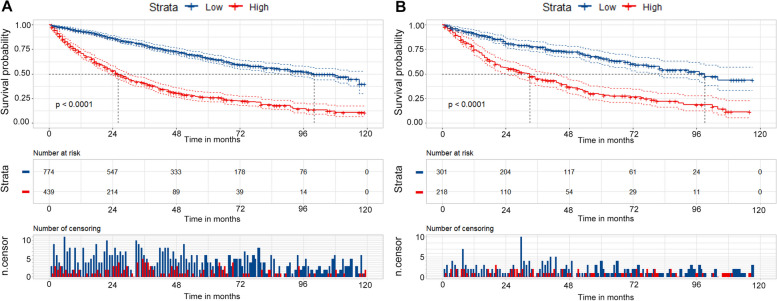
Kaplan–Meier curves of overall survival stratified by risk groups in the **A** training cohort, **B** the validation cohort

Based on the nomogram score for each patient, we classified patients into low-risk (≤ 96) and high-risk (> 96) groups. The Kaplan–Meier survival curves and log-rank tests showed that the high-risk group had a significantly lower survival rate than the low-risk group in both the training and validation sets (*P* < 0.0001) (Fig. 6A, B).

## Discussion

IPM is a heterogeneous category in NSCLC and survival outcomes can be affected by various factors [5, 6, 8]. However, many previous studies have had limited sample sizes and often reported improved survival outcomes due to surgical treatment resulting in downstaging of IPM. Recent research suggests that further downstaging of IPM may be beneficial, emphasizing the importance of continued surveillance of these patients' prognosis [15]. Fortunately, the SEER database contains a wealth of information regarding the treatment and survival outcomes of IPM patients. Leveraging the large sample size provided by the SEER database, we constructed a nomogram that more accurately and conveniently predicts survival outcomes based on patient characteristics.

Our data suggest that patients over 65 years old have a worse prognosis, which is consistent with other studies on lung cancer [20, 21]. However, the prognostic value of age in relation to IPM has been reported differently [5, 6, 8–11]. Older patients may have worse outcomes due to an increased prevalence of comorbidities among lung cancer patients [22–24]. In terms of sex, male patients are widely accepted as having a worse prognosis in other lung cancer studies [25]. Our study found that patients with IPM had poorer survival compared to female patients (HR 1.845, 95% CI 1.558–2.184), which is consistent with previous research [9, 26].

Tumor size is a well-known prognostic factor for NSCLC, and larger tumors have a greater risk of invasion and distant metastasis. In our study, we subdivided primary tumor size into four categories to facilitate statistical analysis by nomogram. We found that primary tumor size was an independent prognostic factor for NSCLC with IPM, with patients having a primary tumor size of ≤ 3 cm having better overall survival (*P* < 0.001). Nakagawa et al. [5] analyzed 48 pathologically diagnosed IPM patients and found that those with tumor sizes > 30 mm had a worse prognosis compared to those with tumor sizes ≤ 30 mm (HR 2.578, 95% CI 1.006–6.608). For PM2, Wang et al. [15] reported that patients with tumor sizes ≤ 3 cm had better overall survival than T4 patients (HR 0.629, 95% CI 0.455–0.869), and their survival outcome was comparable to that of T3 patients. Patients with tumor sizes > 3 cm had a similar survival outcome to T4 patients. However, Ucvet et al. [6] reported that tumor size was not a prognostic factor for survival outcome, which may be due to the fact that 86% of their enrolled patients had tumor sizes of 1 cm or smaller.

The status of lymph node metastases, as reflected by the N status, is a critical prognostic factor for NSCLC. Lee et al. [27] found that patients with N0 had better survival outcomes compared to N1 and N2 patients with IPM. Nakagawa et al. [5] also reported that mediastinal lymph node metastases were a worse prognostic factor for surgically treated patients with IPM. In our study, N status was identified as the most important prognostic factor with the longest line and highest risk score in the nomogram, particularly for N3 in comparison to N0. However, some studies on IPM have reported no negative effects of lymph node involvement. This discrepancy in our findings could be due to the relatively small sample size of these studies [6, 8].

Pulmonary resection with lymph node removal is the standard treatment for NSCLC [28]. Nwogu et al. [29] suggested that resecting more lymph nodes was associated with better patient survival. Samayoa et al. [30] also reported that removing fewer than ten lymph nodes was associated with a 12% increased risk of death in early-stage NSCLC patients. The previous studies concerning IPM needed more relevant information on lymph node removal. Our data showed that removing more regional lymph nodes was associated with better survival in multivariate analysis, highlighting the importance of thoracic lymphadenectomy for IPM.

Tumor histology, specifically the degree of differentiation of tumor tissue, is another important factor affecting prognosis. Sun et al. [31] reported that histologic grade was significantly associated with survival in NSCLC, with undifferentiated carcinoma carrying an 80% increased risk of death compared to well-differentiated carcinoma. Chung et al. [32] found that poorly differentiated tumors had higher lymph node metastasis rates and worse survival compared to well- or moderately differentiated tumors. For NSCLC with IPM, Salazar et al. [11] found that only patients with tumor grade III had statistically significant better survival outcomes than patients with tumor grade I in PM1, while Li et al. [9] found that tumor grade was not a prognostic factor for 3-year OS in PM2 patients. In our study, patients with a histologic grade of well-differentiated were associated with better overall survival.

In terms of treatment for surgically treated patients, chemotherapy is indicated for those with stage II and IIIa non-small cell lung cancer. Salazar et al. [11] investigated the National Cancer Database for NSCLC patients with PM1, including 528 patients, and found that chemotherapy increased 3-year OS. Similarly, Park et al. [10] enrolled 142 patients with PM1 and demonstrated that chemotherapy improved their 5-year OS and disease-free survival, except for patients with tumors smaller than 4 cm. However, Baum documented patients with PM1 from Heidelberg and Berlin databases and suggested that adjuvant therapy did not improve long-term survival if PM1 was the only pT3N0 descriptor [13]. For PM2, Li et al. [9] reported that adjuvant chemotherapy was associated with improved 3-year OS compared to surgery alone. In our study, we found that patients with IPM had significantly improved survival compared to treatment with only surgery (HR 0.805, 95% CI 0.652–0.994, *P* = 0.044).

In our study, we meticulously incorporated several independent prognostic factors, including age, sex, primary tumor size, N status, number of regional lymph nodes removed, tumor grade, and chemotherapy, into the construction of our nomogram. To assess its clinical utility, we rigorously evaluated the nomogram's performance using various metrics such as the C-index, calibration curves, ROC curves, and AUC. These comprehensive evaluations consistently demonstrated that our nomogram exhibits exceptional accuracy, discrimination, and practicality when it comes to predicting overall survival. It's worth noting that recent work by Wang et al. [15] introduced a modified classification for ipsilateral pulmonary metastases, which considers primary tumor size and IPM location. While this approach provides valuable insights, it still relies on the AJCC TNM staging system and may not fully capture the nuances of high-risk patient stratification. Notably, our study takes a pioneering step by introducing the first nomogram tailored specifically for predicting the OS of non-small cell lung cancer patients with IPM. What sets our nomogram apart is its ability to effectively distinguish high-risk patients from low-risk ones based on the total nomogram points. This distinction is underscored by the significant differences in OS observed between these two patient groups in our Kaplan–Meier analysis and log-rank test. With a total nomogram point threshold of 96, clinicians can readily identify high-risk patients among those diagnosed with IPM, facilitating more precise treatment decisions and improved patient care.

Our study has several limitations worth noting. Firstly, due to the retrospective nature of our research, there is a potential for selection bias, and certain crucial variables, such as the size and number of separate tumor nodules, were lacking in the SEER database. It is important to highlight that these variables have not consistently demonstrated significant prognostic associations in the literature [6, 15, 33–35]. Secondly, our study solely relied on data from the SEER database, which may not fully represent the broader population of NSCLC patients with IPM. Therefore, to ensure the generalizability of our nomogram, it is imperative to validate our findings in independent cohorts. Lastly, while our nomogram exhibited strong calibration and discrimination, external validation is indispensable to confirm its performance and clinical utility. Additionally, it's crucial to acknowledge that the SEER database lacks comprehensive data, such as clinical and pathological tumor stages, surgical margins, complete clearance timing of tumors and metastases, specific details regarding the mode, method, and dosage of chemoradiotherapy. Due to insufficient pathological data, we were unable to study the postoperative number of positive lymph nodes and pN. Furthermore, data pertaining to genetic testing and immunotherapy, which are relevant to cancer patient prognosis [36–38], remain incomplete. Despite these limitations, our study offers a valuable tool for predicting survival outcomes in NSCLC patients with IPM, relying on readily available clinical factors.

## Conclusion

In conclusion, age, sex, primary tumor size, N status, number of regional lymph nodes removed, tumor grade, and chemotherapy of patients were the independent prognostic factors for NSCLC patients with IPM. We constructed and internally validated a nomogram to predict 1-year, 3-year, and 5-year OS for NSCLC patients with IPM according to independent prognostic factors. After internal validation, this novel nomogram demonstrated good calibration, discrimination, clinical utility, and practical decision-making effects for the prognosis of NSCLC patients with IPM.

## Data Availability

All data and documents needed will be provided upon request through email: 361532004@qq.com.

## Abbreviations

NSCLC: Non-small cell lung cancer
OS: Overall survival
IPM: Ipsilateral pulmonary metastasis
AJCC: American Joint Committee on Cancer Staging
TNM: Tumor-node-metastasis
SEER: Surveillance, Epidemiology, and End Results
SCC: Squamous cell cancer
ADC: Adenocarcinoma
PM1: Tumors with additional nodule(s) in the same lobe
PM2: Tumors with additional nodule(s) in the ipsilateral different lobe
